# Concussion in University Level Sport: Knowledge and Awareness of Athletes and Coaches

**DOI:** 10.3390/sports6040102

**Published:** 2018-09-20

**Authors:** Ben Kirk, Jamie N. Pugh, Rosanna Cousins, Shaun M. Phillips

**Affiliations:** 1 School of Health Sciences, Liverpool Hope University, Hope Park, Liverpool L16 9JD, UK; cousinr@hope.ac.uk; 2 Research Institute for Sport and Exercise Sciences, Liverpool John Moores University, Liverpool L3 5UA, UK; j.pugh@2014.ljmu.ac.uk; 3 Institute for Sport, Physical Education & Health Sciences, University of Edinburgh, Scotland EH8 8AQ, UK; shaun.phillips@ed.ac.uk

**Keywords:** sport-related concussion, signs, symptoms, return to play guidelines, traumatic brain injury

## Abstract

Using a cross-sectional survey concussion knowledge was evaluated among forty university-level athletes (*n* = 20, rugby union players; *n* = 20, Gaelic football players) and eight experienced team coaches (*n* = 2, rugby union; *n* = 2, Gaelic football; *n* = 1, soccer; *n* = 1, hockey; *n* = 1, netball; *n* = 1, basketball). Levels of knowledge of concussion were high across all participants. Coaches had higher knowledge scores for almost all areas; however, there was evidence of important gaps even in this group. Knowledge was not sufficient in identifying concussion, and when it is safe to return to play following a concussion. Impaired knowledge of how to recognise a concussion, and misunderstanding the need for rest and rehabilitation before return to play presents a hazard to health from second impact and more catastrophic brain injury. We discuss reasons for these guideline misconceptions, and suggest that attitude issues on the significance of concussion may underlie a willingness to want to play with a concussion. This suggests the current education on sport-related concussion needs to be expanded for the appropriate management of university-level contact sports.

## 1. Introduction

Concussion, a type of traumatic brain injury (TBI), can occur following a forceful impact to the head, face, neck, or body that induces sudden impulsive trauma to the brain. The American Medical Society for Sports Medicine defines concussion as a “transient disturbance of brain function” [1]; the UK online National Health Service similarly asserts that concussion is usually a temporary injury [2]. However, there is evidence that the physiological changes that follow concussion provide potential for chronic, as well as acute, physical, cognitive, and emotional impairments [3,4]. It follows from this that concussion is an underappreciated public health issue [5] that presents a serious situation with possible long-term challenges to health.

Concussion should be suspected whenever there are changes in mental status following impact on a sports field [6]. This requires an immediate decision on whether an occasion where there has been forceful impact was sufficient to cause concussion. Early observable features of concussion include headache, disorientation, vomiting, nausea, dizziness, slurred speech, and delayed responses [6,7]. There may be temporary loss of consciousness, although this is not necessary to suspect concussion [7].

Players of contact sports are at risk of concussion [5,8]. This raises the question of sufficiency of knowledge of concussion in this population, particularly in amateur university-level team sports, where medical support is generally not on site. The question is becoming more critical in view of accumulating evidence of symptoms of concussive brain injury such as persistent headaches, confusion, irritability, sleep disturbance, amnesia, and fatigue continuing for several months, pointing to more long-term negative health effects of concussion. Additionally, brain function studies in former athletes and post-mortem have provided evidence that even when asymptomatic in youth, cognitive and motor impairments can become apparent in later life [9,10,11]. Most recently, a retrospective population study reported an elevated risk of early-onset dementia in those with a history of moderate to severe TBI [12].

It is known that the incidence of concussion in youth sports is not negligible [5,7]. For example, 5.1% of the sample of 17,659 collegiate and high school football players in the USA sustained at least one concussion during play in a single season, and that 14.7% of this number suffered a second concussion during the same season [7], potentiating severity [13]. Although based on a much lower sample size, findings from an incidence study [14] of Rugby union players in Ireland under 20 years old were that 64 of the 133 reported they had experienced at least one concussion in their playing history. Of these players, 61 reported their symptoms to their coach; however, just 36 (56%) sought medical attention, in line with previous findings of underreporting concussions and low adherence to return to play guidelines [15,16,17,18,19,20]. Primary reasons cited by athletes for failing to report their concussion are: not thinking the injury was serious enough (63%), not wanting to leave the field of play (41%), and being unaware they had suffered a concussion (36%) [14].

Medical underreporting of concussions by athletes is a major concern due to the potentially serious consequences of the injury. It also raises questions regarding knowledge of concussion in high-risk populations. Most research in this area has been undertaken in the United States of America (USA). In response to this research, the Centres for Disease Control and Prevention (CDC) launched the “Heads Up” campaign in 2003 [21] to provide information for high school and youth sports team coaches. The intervention materials were widely distributed in the USA; however, it has been suggested that although initial gains in knowledge led to increased efforts to minimise risks associated with concussion, the intervention has not stood the test of time [22]. There has been no obvious promulgation of the “Heads Up” information materials across the Atlantic Ocean, raising the question of whether there is a need to improve knowledge of short-time and long-term sequelae of concussions in amateur sport in the UK. A primary starting point is to ascertain what is already known.

Investigating knowledge of concussion is justified as many thousands of young people participate in impact sports—particularly at the university level. Rugby union and Gaelic football are popular contact sports in universities, yet there is no literature on levels of knowledge of concussion in players or their coaches. This research was undertaken to address that fact, in the first instance, towards supporting a risk assessment of concussion in this setting. Coaches were included as well as players, as coaches are often the first individual to recognise that an athlete could have a concussion and, importantly, in amateur university-level sports, coaches typically have little to no education on concussion [23]. In line with participation in the contact sports used in this study, rugby union and Gaelic football, the participants were all male. On the basis of the literature, it was anticipated that misconceptions in concussion knowledge would be evident in coaches and athletes in both sports.

## 2. Materials and Methods

### 2.1. Subjects

Following institutional ethical approval (ID: 1007305), *n* = 20 rugby union players (age: 22 ± 2 years, playing experience: 8 ± 1 years), *n* = 20 Gaelic football players (age: 21 ± 1 years; playing experience: 5 ± 1 years), and *n* = 8 coaches (age: 28 ± 2 years; coaching experience: 6 ± 1 years) were recruited from university contact sports teams. Coaches were recruited from Gaelic football, rugby union, soccer, hockey, netball, and basketball to increase the limited sample of contact sports coaches. All participants were male and competing in amateur university leagues.

### 2.2. Procedure

Recruitment was carried out via email circulated to university sports personnel to enquire if coaches and respective players would like to take part in a survey concerning an “aspect of health”. All participants who expressed an interest provided written informed consent and then completed the same survey prior to a team training session individually in a private room with only the researcher present. This prevented the possibility of participants viewing each other’s responses and/or conferring with one another.

### 2.3. Survey

The previously validated pre-test questionnaire from the CDC Concussion in Youth Sports campaign was utilised [21]. This questionnaire comprised 11 items that assess knowledge of concussion, including items related to mechanisms, signs, and symptoms of concussion and return to play guidelines. Each item had three alternatives, and participants could choose what they believe to be the correct response. Correct answers scored a value of 1, and incorrect responses scored a value of 0. This provided a final score for each participant between 0 and 11; knowledge across items was also assessed, and the sum of correct responses according to type of participant was expressed as a percentage for analysis.

### 2.4. Statistical Analysis

SPSS Statistics 22 (IBM, Chicago, IL, USA) was used to analyse the data. The alpha level for statistical significance was set at *p* < 0.05. All variables were tested for normality (Shapiro-Wilk test) and homogeneity of variance (Levene’s test). A one-way analysis of variance was used to establish differences between groups. Significant main effects were followed up using Tukey’s post-hoc analysis.

## 3. Results

As can be seen in Table 1, knowledge of concussion levels in university-level players and coaches was quite high overall; nevertheless, there were some important misconceptions—particularly surrounding return to play after a concussion and identifying concussion. To test whether the observed difference in means between groups (Rugby Union, Gaelic Football, Coaches) was statistically significant, we performed a one-way analysis of variance. There was a significant main effect for group (F = 8.22, *p* < 0.01); post-hoc analyses demonstrated a significantly higher proportion of correct responses from coaches in comparison to Gaelic football players (*p* < 0.01). Whilst approaching significance (*p* = 0.068), there was no significant difference in knowledge between coaches and rugby union players, and there was no difference in concussion knowledge between Gaelic football and rugby union players.

With respect to coaches, we found all could identify the signs of a concussion, and most (95%) were correct in identifying when concussion can occur. All coaches were aware of the procedures to follow in the event of a concussion and that they should tell parents immediately. In contrast to guidelines, however, 25% of coaches believed that an athlete could return to play immediately after being evaluated by a health care professional.

With respect to players, 40% of Gaelic footballers were unaware that a concussion was identified by watching for a change in the athlete’s behaviour, thinking, or physical functioning. Similarly, 25% of Gaelic footballers were not aware that “if the athlete appears stunned, is unsure of the game, score, or opponent, is confused about their assignment or position, and is answering questions slowly” they may be suffering from a concussion. For both rugby union players and Gaelic footballers, 20% had incorrect beliefs of what a concussion is and the same proportion considered that organisers should “allow an athlete to finish the game and then seek medical attention”. Additionally, 35% of rugby union players and 55% of Gaelic footballers did not know that organisers should not allow an athlete to return to play as soon as they are feeling better.

## 4. Discussion

Knowledge of concussion in our sample of university-level sportsmen was insufficient in various areas. Coaches scored better than players on almost all aspects of concussion, and their knowledge was significantly superior to Gaelic football players. There were, nevertheless, important gaps in knowledge of concussion even in coaches, even when asked using a forced-choice response format, which is less demanding than generating answers from memory. Our results corroborate previous findings [18,24] that athletes and coaches across various sports hold misconceptions surrounding concussion. Misunderstandings were particularly pertinent in the two questions asking about identifying a concussion and returning to play after a concussion. This presents a hazard to health for the large numbers known to engage in contacts sports at university, and indicates intervention towards improving knowledge.

Regarding identifying a concussion, it is important for players and coaches to know that the hallmarks of concussion are confusion and amnesia [6], and that these can be recognised on the field and in the community. Nearly one-quarter of coaches in this study considered that “looking at scans of an athlete’s brain” was the way to diagnose a concussion. However, they are not alone in their mistake: a similar study found that 50% of Italian soccer coaches identified brain scans as the primary method to detect concussions [17].

The majority of players, and all coaches, knew that after a blow to the head or body an athlete must be immediately removed from play to look for symptoms of concussion; the athlete must not be allowed to play on before taking a medical examination. This is reassuring, in that even though one fifth of players, a similar figure to previous studies [15,19,24], believed it was acceptable to continue playing while experiencing concussion symptoms, a knowledgeable coach would remove them from play. Linked to this, more than 90% of the sample understood that a history of concussion increases the likelihood of a recurring injury.

From our data, we found most errors in knowing when it is safe for an athlete to return to play. All athletes who have experienced concussion should have a medical examination [25], and it is this that should determine the extent of need for rest and rehabilitation according to severity. Return to play is determined by a six-step procedure and a player must be asymptomatic before being allowed to return to play [25,26]. In our study, not all coaches had knowledge of these guidelines; similarly, just under half of players were aware of the mandatory return to play guidelines [26]. It has previously been reported that 40% of high school athletes return to play prematurely after a reported concussion [16], indicating there may a fundamental attitude problem underlying this misconception.

Return to play guidelines are an important part of tackling the negative impact of sport-related concussion. Warnings of the susceptibility for a second impact changing a relatively minor concussion into a catastrophic brain injury have been in the literature since at least 1984 [13], accompanied by authoritative guidelines on management of concussion [6] and return to play [25]. Nevertheless, our findings demonstrate that a substantial number of athletes perceive it to be safe to return to play as soon as they feel better.

Failing to follow return to play guidelines can be explained in two ways: by lack of knowledge of the guidelines and by an imprudent attitude towards the consequences of concussion. With respect to knowledge, there have been various campaigns [21,22,27] to improve concussion education. Nevertheless, high rates of concussion in contact sports continue to be reported. It has been noted that there has been limited research supporting the effectiveness of these programmes [23,28]. In particular, a prospective study of concussion education in university-level ice hockey players in the USA found no significant improvements in knowledge and called into question the education process [28]. It is plausible that athletes who have been educated on concussion have not assimilated the information, because it is at odds with their attitudes to concussion and sporting behaviour. That is, premature return to play is due to attitudes that encompass a desire to succeed, not wanting to let the team down, and other similar pressures [24,27,29]. Such possibilities can be satisfactorily explained with reference to cognitive dissonance theory [30]. To illustrate: consider that those voluntarily involved in university-level contact sports feel that playing football/rugby, etc. is enjoyable, and they believe that participation is an important support for their health and wellbeing. Thus, their behaviour on the field is that they fully engage with all aspects of the game. If then, as a player or as a coach, they are presented with an injury on the field, the belief that they should stop play is a threat to their more heavily reinforced belief that playing is good. That is, the person has two opposing cognitions: playing is good and stopping play now is good. Since these two cognitions cannot be reconciled, one has to change. Studies on attitude have found that once formed they are resistant to change, which can explain why the cognition “playing is good” may prevail unless the magnitude of dissonance is sufficiently raised by an affective appreciation of “stopping play now is good”, the latter of which would be more beneficial to them regardless of any negative impact on the game and team.

It follows from this that modes of education around concussion require more than presenting bald facts and directions; a consideration of existing attitudes and an understanding that they are ultimately based upon the social norms of their team sport are also necessary. Social norms are learned, socially-based rules that prescribe behaviour in a given situation; on the sports field it is that one fully engages in the team game. Stopping play also presents a challenge to behaving in line with given social norms, as well as one’s own attitude and unrealistic optimism about their own comparative level of risk.

## 5. Conclusions

In conclusion, whilst appreciating the limitations of this study in terms of sample type and size, the findings reported here illustrate that there are misconceptions in knowledge of concussion among university-level athletes and coaches. We provide a robust, theory-based explanation for the persistently high levels of inappropriate reaction to sport-related concussion [20]. The recommendations that flow from the explanation is for improved educational tools that take into account existing attitudes to benefits and risks in playing contact sports; this can be generalised to other groups playing contact sports. Perhaps we can learn from previous social norms media marketing strategies successful at changing health behaviours with respect to tobacco usage [31], drink driving [32], and sugar consumption [33]. In turn, this may reduce the prevalence of TBI.

## Figures and Tables

**Table 1:** Percentage of correct group responses to the Centres for Disease Control and Prevention (CDC) Concussion in Youth Sport Questionnaire (abridged here).

Question	Correct Responses of Participants Based on Group (%)		
	Rugby Union (*n* = 20)	Gaelic Football (*n* = 20)	Coaches (*n* = 8)
1. A concussion is a…	80	80	100
2. When can concussions occur?	85	80	95
3. How do you identify a concussion?	85	60	75
4. Which are signs of a concussion?	95	75	100
5. Which are symptoms of a concussion?	95	85	100
6. What are the consequences of a previous concussion?	90	90	95
7. What is the first thing to do when player has sustained a blow to the head and is not acting right?	80	80	100
8. Which are signs of a severe concussion and requiring emergency treatment?	85	80	95
9. When can an athlete return to play after a concussion?	65	45	75
10. When should an athlete’s parents know about the possible concussion?	85	80	100
11. How can you help prevent concussions?	70	75	95
Mean score (SD)	83.18 (9.29)	75.45 (12.54)	93.64 (9.51)
